# Machine Learning Analysis of Lipid and Metabolic Profiles in Adults with Adenoid Hyperplasia

**DOI:** 10.3390/medicina61061018

**Published:** 2025-05-29

**Authors:** Mansur Doğan, Merve Çiftçi, Yusuf Yeşil

**Affiliations:** 1 Department of Otorhinolaryngology, Faculty of Medicine, Sivas Cumhuriyet University, Sivas 58140, Türkiye; 2 Department of Otorhinolaryngology, Tokat Erbaa State Hospital, Tokat 60500, Türkiye; mervekocciftci@gmail.com; 3 Department of Biochemistry, Tokat Erbaa State Hospital, Tokat 60500, Türkiye; dryesilyusuf@gmail.com

**Keywords:** lipid profiles, adults, adenoid hyperlasia

## Abstract

*Background and Objectives*: The nasopharynx, unlike other pharyngeal regions, includes an important part of the immune system, called the adenoid (nasopharyngeal tonsil); its posterior wall contains lymphoid tissue belonging to Waldeyer’s ring. Nasopharyngeal posterior wall thickness is often associated with adenoid hyperplasia in adults. The current study aimed to compare the blood lipid and metabolic profiles of adult patients with increased nasopharyngeal posterior wall thickness to those of the healthy population. *Materials and Methods*: This study included a cohort of 98 patients, 52 in the control group and 46 diagnosed with increased nasopharyngeal posterior wall thickness due to adenoid hyperplasia. Clinical and biochemical data were collected from medical records at Sivas Cumhuriyet University and Erbaa State Hospital between January 2024 and March 2025. The dataset consisted of the following 11 features: age, sex, total cholesterol, low-density lipoprotein (LDL), high-density lipoprotein (HDL), triglycerides, fasting blood glucose, glycated hemoglobin (HbA1C), C-reactive protein (CRP), alanine aminotransferase (ALT), and aspartate aminotransferase (AST). *Results*: HDL was significantly lower in the adenoid hyperplasia group (mean = 48.68, SD = 21.87) compared to the control group (mean = 51.31, SD = 11.80; Kruskal–Wallis H = 4.750, *p* = 0.029), with a small effect size (Cohen’s d = −0.156). ALT was higher in the adenoid hyperplasia group (mean = 26.35, SD = 16.93 vs. 20.88, SD = 11.42; permutation test *p* = 0.082), suggesting a trend toward significance. HbA1C had a higher mean in the adenoid hyperplasia group (7.88, SD = 9.82 vs. 6.18, SD = 1.18; *p* = 0.852), with high variability. *Conclusions*: In conclusion, this study identified HDL, HbA1C, and ALT as potential biomarkers for nasopharyngeal adenoid hyperplasia, with XGBoost and SHAP providing valuable insights despite dataset constraints.

## 1. Introduction

The nasopharynx refers to a fibromuscular area on the soft palate behind the nasal cavity [1]. The nasopharyngeal posterior wall is a special area containing tissue known as the adenoid (nasopharyngeal tonsil), which encompasses lymphoid tissues belonging to Waldeyer’s ring [2]. Nasopharyngeal posterior wall thickness, often associated with adenoid hyperplasia in adults, is an uncommon finding that may signal underlying pathophysiological processes beyond malignancy [3]. The adenoids typically reach their maximum size in childhood and undergo involution by adolescence [4]. However, an increased nasopharyngeal posterior wall thickness persists or develops in some adults, potentially contributing to mechanical upper airway obstruction and chronic nasopharyngeal inflammation symptoms such as nasal congestion, snoring, and rhinosinusitis [5]. The mechanisms that drive this hyperplasia, particularly its relationship with metabolic and lipid profiles, are still poorly understood.

An increased nasopharyngeal posterior wall thickness due to adenoid hyperplasia can lead to significant clinical consequences, including chronic inflammation or infection, which may interact with metabolic pathways. Previous research has suggested that upper airway obstruction, such as adenoid hypertrophy in children, is associated with altered lipid profiles, notably reduced HDL levels, potentially linked to obstruction severity [6]. Whether similar relationships exist in adults with persistent nasopharyngeal adenoid tissue is unclear. Lipid dysregulation, including low HDL and elevated triglyceride levels, is a known risk factor for cardiovascular diseases, and its potential connection to nasopharyngeal adenoid pathology can provide novel clinical insights [7]. Furthermore, metabolic markers such as HbA1C and liver enzymes (ALT, AST, and GGT) may reflect systemic changes linked to chronic inflammation or oxidative stress, leading to the hypothesis of a broader metabolic impact of nasopharyngeal obstruction [8].

Magnetic resonance imaging (MRI) is a critical tool for assessing nasopharyngeal posterior wall thickness with high soft tissue resolution. On MRI images, nasopharyngeal adenoid hyperplasia is typically a symmetric mass that is slightly hyperintense on T2-weighted images, isointense on T1-weighted images, and shows slight enhancement after contrast material [9]. Vogler et al. demonstrated that the adenoids decrease in size with age, reaching their largest size in the 7–10-year age group (14.59 mm on average) and decreasing to 4.83 mm at 60 [4]. In our study, we accepted the threshold value for nasopharyngeal posterior wall thickness as 5 mm, taking the study by Vogler et al. as a reference. Another study accepted the pathological limit as 3 mm [10].

In a study conducted in Taiwan, the effect of a biofilm layer on adenoid hypertrophy was examined when adenoid hyperplasia was detected in children. It was revealed that chronic inflammatory processes would be effective in adenoid hyperplasia [11]. In another study, the relationship between adenoid hypertrophy and laryngopharyngeal reflux was evaluated in 30 children, and a direct relationship was found [12]. In our study, we wanted to investigate whether blood lipid and metabolic parameters would change significantly in lymphoid tissue hyperplasia in response to chronic inflammatory processes in adult patients.

In the present work, our objective is to understand the relationship between nasopharyngeal posterior wall thickness and lipid metabolism in adults, as well as to guide the assessment of cardiovascular risk factors and the development of novel approaches in patient management. In this way, we aim to provide an interdisciplinary contribution to both otolaryngology and endocrinology.

## 2. Materials and Methods

### 2.1. Study Population and Data Collection

This study included a cohort of 98 patients, 52 in the control group and 46 diagnosed with increased nasopharyngeal posterior wall thickness due to adenoid hyperplasia. Clinical and biochemical data were collected from medical records at Sivas Cumhuriyet University and Erbaa State Hospital between January 2024 and March 2025. The dataset consisted of the following 11 features: age, sex (encoded as 0 for females and 1 for males), total cholesterol, LDL, HDL, triglycerides, fasting blood glucose, HbA1C, CRP, ALT, and AST. The target variable was the status of nasopharyngeal adenoid hyperplasia (0 for control and 1 for adenoid persistence).

Patients with adenoid hyperplasia were identified through MRI. Adenoid hyperplasia was defined as nasopharyngeal posterior wall thickness > 5 mm, per [4], and clinical symptoms such as nasal congestion, snoring, or rhinosinusitis, with at least a partially complete biochemical profile (including total cholesterol, LDL, HDL, triglycerides, fasting blood glucose, HbA1C, CRP, ALT, or AST). The control group comprised individuals with no adenoid hyperplasia (nasopharyngeal posterior wall thickness ≤ 5 mm on MRI), no upper airway obstruction symptoms, no history of nasopharyngeal pathology, and similar biochemical data availability. The exclusion criteria for both groups included severe hepatic or renal dysfunction, active malignancy, autoimmune diseases, systemic infections, incomplete records, pregnancy, lactation, or refusal to participate. The adenoid hyperplasia group was further excluded for prior adenoidectomy, head/neck radiotherapy, or craniofacial anomalies, while controls were excluded for chronic nasopharyngeal or upper airway pathology or prior nasopharyngeal surgery. Several features had missing data, notably HbA1C (n = 83 total; 34 for adenoid hyperplasia), HDL (n = 92; 40 for adenoid hyperplasia), etc., with sample sizes ranging from 83 to 98 across features.

### 2.2. Data Preprocessing

Data were preprocessed using Python 3.8 with the pandas and scikit-learn libraries. To assess missingness patterns, we evaluated whether data were Missing Completely at Random (MCAR), Missing at Random (MAR), or Missing Not at Random (MNAR). Little’s MCAR test was applied, yielding a non-significant result (*p* > 0.05), suggesting that missingness was not entirely random. Logistic regression analyses examined associations between missingness in HbA1C and HDL and the observed variables (e.g., age, sex, adenoid hyperplasia status), revealing weak correlations with age and group status (*p* < 0.10), indicative of MAR. MNAR was considered possible for HbA1C, as missingness might relate to unrecorded diabetes status, but this could not be directly tested. Given the clinical context and small sample size, we assumed MAR for imputation purposes. Missing values were imputed using the median strategy to maintain robustness against non-normal distributions, as confirmed by Shapiro–Wilk tests (e.g., HDL, HbA1C, and ALT were classified as non-normal). The dataset was split into training (80%, ~78 samples) and testing (20%, ~20 samples) using stratified sampling to preserve the class distribution (approximately 53% control and 47% adenoid hyperplasia). Thus, a balanced representation of both groups was ensured.

### 2.3. Statistical Analysis

Descriptive statistics, including means, standard deviations, and sample sizes, were calculated for each feature. Normality was assessed using the Shapiro–Wilk test. Group differences were evaluated using ANOVA for normally distributed features (reporting F-statistics and *p*-values), the Kruskal–Wallis test for non-normal features (reporting H-statistics and *p*-values), and permutation tests with 1000 permutations for robust *p*-values. A *p*-value threshold of <0.05 was used to determine statistical significance across all tests. Cohen’s d was calculated as an effect size measure, defined as the mean difference between groups divided by the pooled standard deviation. Group differences were evaluated using multiple statistical tests to account for distributional properties:

- ANOVA: applied for normally distributed features or as a complementary test, with results reported as F-statistics and *p*-values.
- Kruskal–Wallis test: used for non-normal features, reporting H-statistics and *p*-values.
- Permutation test: conducted with 1000 permutations to confirm findings, reporting *p*-values robustly.
- Cohen’s d: calculated as an effect size measure, defined as the mean difference between groups divided by the pooled standard deviation.

### 2.4. Machine Learning Model Development

An eXtreme Gradient Boosting (XGBoost) classifier was developed to predict the status of nasopharyngeal adenoid hyperplasia, leveraging its capability to model non-linear relationships in small datasets. The model was implemented using the XGBoost library in Python. The hyperparameters were configured as follows: n_estimators = 100, max_depth = 3, learning_rate = 0.1, random_state = 42, and eval_metric = ‘logloss’. To address the slight class imbalance (scale_pos_weight = 1.13, calculated as the ratio of control to adenoid hyperplasia samples), the scale_pos_weight parameter was applied to penalize the misclassification of the minority class.

The model was trained on the imputed training set and evaluated on the testing set. Performance metrics included accuracy, precision, recall, F1-score, and area under the receiver operating characteristic curve (ROC-AUC). Five-fold cross-validation was performed to assess model stability, reporting mean ROC-AUC and standard deviation.

### 2.5. Model Explainability

SHapley Additive exPlanations (SHAP) values were computed using the SHAP library to enhance interpretability. A TreeExplainer was applied to the trained XGBoost model to calculate SHAP values for all features across the dataset. Two visualizations were generated:

- A bar plot displaying mean absolute SHAP values, ranking features by their average impact on predictions.
- A summary plot illustrating feature contributions, with colors indicating whether high or low feature values increased the likelihood of adenoid hyperplasia (red) or control (blue).

Feature importance was also extracted from the XGBoost model, based on gain, to complement SHAP analysis and identify the key predictors.

### 2.6. Model Evaluation and Visualization

Model performance was evaluated on the testing set, yielding the following: accuracy = 0.650, precision = 0.625, recall = 0.556, F1-score = 0.588, and ROC-AUC = 0.626. Cross-validation produced a mean ROC-AUC of 0.669 (standard deviation = 0.104), indicating moderate discriminative ability with variability attributed to the small sample size. Feature importance identified HDL (0.178), HbA1C (0.138), and fasting blood glucose (0.110) as the top predictors, while sex had a negligible impact (0.000).

The following visualizations were generated using Matplotlib (v3.10.0) and Seaborn (v0.13.2):

- An ROC curve plotting the true-positive rate against the false-positive rate, annotated with the AUC.
- A feature importance bar plot ranking the features by XGBoost gain.
- A SHAP bar and summary plots for model explainability.
- Box plots comparing the distributions of significant or near-significant features (HDL, ALT, total cholesterol, and HbA1C) between the groups, saved as PNG files.

### 2.7. Software and Statistical Tools

Analysis was conducted in Python (version 3.8) using the following libraries: pandas (version 1.5.3) for data manipulation, scikit-learn (version 1.2.2) for preprocessing and metrics, XGBoost (version 1.7.3) for modeling, SHAP (version 0.42.1) for explainability, Matplotlib (version 3.7.1) and Seaborn (version 0.12.2) for visualization, and NumPy (version 1.24.3) and SciPy (version 1.10.1) for statistical computations. The code was executed in a Jupyter Notebook environment (v7.1.0) for reproducibility.

## 3. Results

Descriptive statistics revealed differences between the groups (Table 1 and Table 2). HDL was significantly lower in the adenoid persistence group (mean = 48.68, SD = 21.87) compared to the control group (mean = 51.31, SD = 11.80; Kruskal–Wallis H = 4.750, *p* = 0.029), with a small effect size (Cohen’s d = −0.156). ALT was higher in the adenoid persistence group (mean = 26.35, SD = 16.93 vs. 20.88, SD = 11.42; permutation test *p* = 0.082), suggesting a trend toward significance. Total cholesterol was lower in the adenoid persistence group (mean = 166.67, SD = 56.23 vs. 182.35, SD = 42.85; *p* = 0.128), albeit insignificantly. HbA1C had a higher mean in the adenoid persistence group (7.88, SD = 9.82 vs. 6.18, SD = 1.18; *p* = 0.852) with high variability. The other features, including LDL (*p* = 0.858), triglycerides (*p* = 0.834), fasting blood glucose (*p* = 0.332), CRP (*p* = 0.445), and AST (*p* = 0.718), displayed no significant differences. After a Bonferroni correction was applied for 30 tests (10 biomarkers × 3 tests, α’ = 0.05/30 = 0.00167), no biomarker achieved statistical significance, as HDL (*p* = 0.029) and ALT (*p* = 0.082) exceeded the adjusted threshold. However, HDL remained the closest to significance, with a notable effect size, suggesting its potential relevance pending further validation. A power analysis was conducted for a two-sample *t*-test using this study’s sample sizes (46 adenoid hyperplasia, 52 control) at 80% power and α = 0.05 (two-tailed). The analysis indicated that this study was powered to detect a Cohen’s d of approximately 0.57, suggesting sufficient sensitivity for moderate effect sizes but limited power for smaller effects. The small effect size for HDL and its wide confidence interval, which includes zero, suggest that the pre-correction statistical significance may have limited clinical impact, though its prominence in predictive modeling warrants further exploration.

### 3.1. Distribution of Parameters

**Figure 1:**
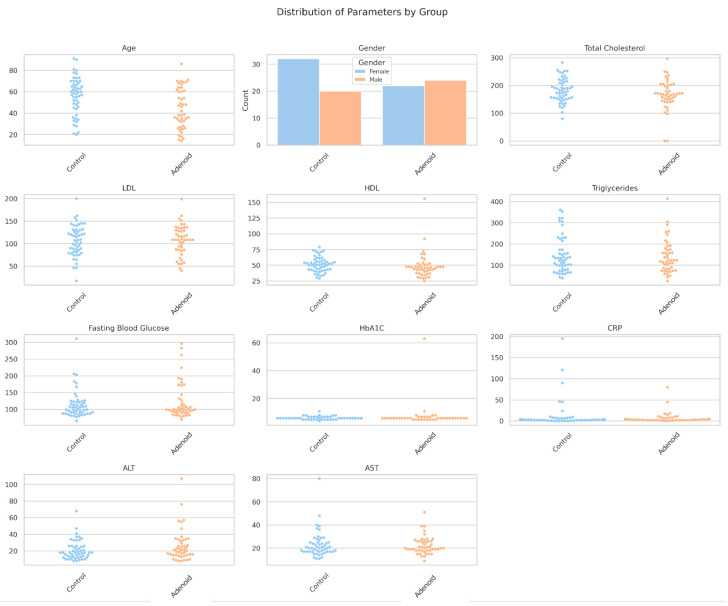
Distribution of biochemical parameters by group. A logistic regression model with HDL and sex as predictors was not significant (HDL: coefficient = −0.107, *p* = 0.915; sex: coefficient = 0.138, *p* = 0.890), indicating limited linear predictive power.

A single figure was generated to visualize the distribution of all parameters using swarm plots for continuous features and a count plot for sex (Figure 1). HDL showed a clear separation, with lower values in the adenoid persistence group, consistent with its statistical significance (*p* = 0.029). ALT exhibited higher values in the adenoid persistence group, which supported the near-significant trend (*p* = 0.082). HbA1C displayed higher values in the adenoid persistence group, albeit with fewer data points (n = 83) and high variability. Total cholesterol shifted downward slightly in the adenoid persistence group, while the other features (e.g., LDL and triglycerides) overlapped considerably. Sex distribution was similar between the groups, with no notable differences in female-to-male ratios.

### 3.2. XGBoost Model Performance

**Figure 2:**
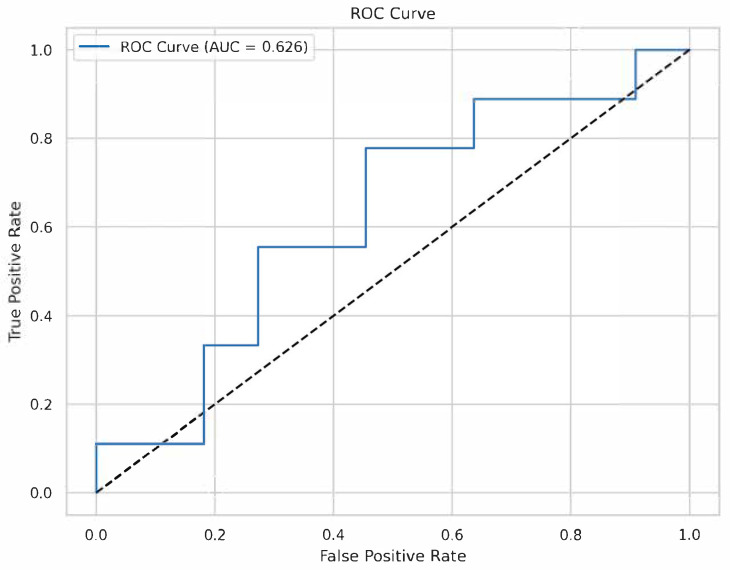
Receiver operating characteristic (ROC) curve. The ROC curve for the XGBoost model on the testing set, with an AUC of 0.626.

The XGBoost classifier, trained on 80% of the data (~78 samples) and tested on 20% (~20 samples), achieved moderate performance: accuracy = 0.650, precision = 0.625, recall = 0.556, F1-score = 0.588, and ROC-AUC = 0.626 (Figure 2). Five-fold cross-validation yielded a mean ROC-AUC of 0.669 (SD = 0.104), indicating variability, likely due to the small sample size. The model’s scale_pos_weight was set to 1.13 to account for the slight class imbalance (52 control, 46 adenoid persistence).

### 3.3. Feature Importance and SHAP Analysis

**Figure 3:**
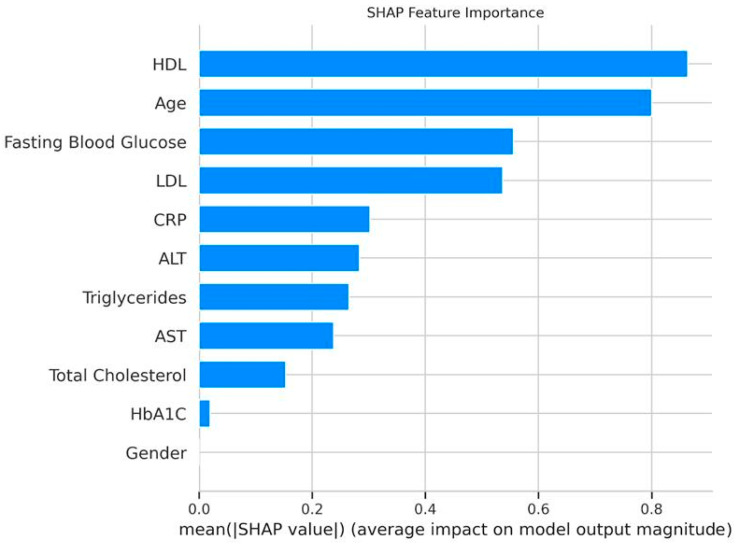
XGBoost feature importance. Bar plot ranking features by their contribution to the XGBoost model, with HDL (0.178) as the top predictor.

Feature importance from the XGBoost model identified HDL (0.178), HbA1C (0.138), and fasting blood glucose (0.110) as the top predictors, followed by age (0.103), LDL (0.097), AST (0.088), CRP (0.082), ALT (0.078), triglycerides (0.067), and total cholesterol (0.060) (Figure 3). Sex had no predictive contribution (0.000), which agrees with the logistic regression results.

**Figure 4:**
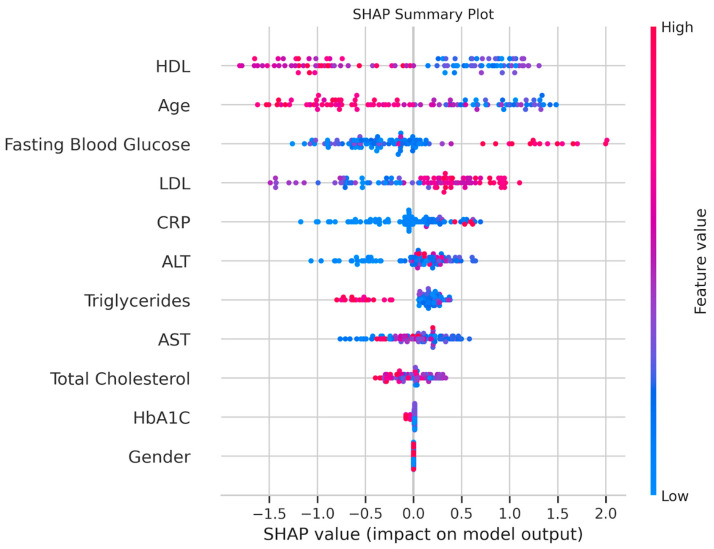
SHAP analysis. Plot showing feature contributions, with red indicating an increased probability of adenoid persistence and blue indicating the control.

SHAP analysis provided further interpretability (Figure 4). The SHAP bar plot confirmed HDL, HbA1C, and fasting blood glucose as the top predictors. The SHAP summary plot revealed that lower HDL values were associated with a higher probability of nasopharyngeal carcinoma, which aligned with the statistical findings. Higher HbA1C values increased the likelihood of nasopharyngeal carcinoma, despite its insignificant *p*-value, suggesting a non-linear predictive power. Elevated ALT and fasting blood glucose values also contributed positively to nasopharyngeal carcinoma predictions, whereas sex had a minimal impact.

### 3.4. Key Observations

The combination of statistical and machine learning analyses highlighted HDL as a significant biomarker (*p* = 0.029, top feature at 0.178), with lower values in the adenoid persistence group. HbA1C emerged as a notable predictor in the XGBoost model (0.138), despite high variability and non-significance (*p* = 0.852), which suggested potential glycemic dysregulation. ALT showed a near-significant trend (*p* = 0.082) and moderate model contribution (0.078), indicating possible liver involvement. The moderate ROC-AUC (0.626) and high cross-validation variability (SD = 0.104) underscored limitations due to the small sample size and missing data (e.g., HbA1C n = 83).

## 4. Discussion

In this study, we utilized an XGBoost classifier to predict the status of nasopharyngeal adenoid persistence in a cohort of 98 patients and identified HDL, HbA1C, and fasting blood glucose as the top predictors, with HDL demonstrating both statistical significance (*p* = 0.029) and the highest model importance (0.178). The model achieved a moderate ROC-AUC of 0.626, with cross-validation indicating variability (mean = 0.669, SD = 0.104), which was possibly due to the small sample size and missing data. These findings, supported by SHAP analysis and parameter distribution visualizations, provide insights into potential biomarkers for nasopharyngeal adenoid hyperplasia, despite limitations that warrant cautious interpretation.

The significant association of lower HDL levels with adenoid hyperplasia (mean = 48.68 vs. 51.31, *p* = 0.029, Cohen’s d = −0.156) aligns with evidence linking lipid dysregulation to upper airway obstruction. HDL, known for its role in cholesterol efflux and its anti-inflammatory properties, may reflect altered metabolic states in adenoid persistence, potentially influencing chronic inflammation dynamics. Previous studies have reported reduced HDL levels in adenoid hypertrophy in children, suggesting a parallel in adults [6]. The prominence of HDL in both statistical tests and the XGBoost model underscores its potential as a biomarker, warranting further investigation into HDL subtypes (e.g., HDL-C and apolipoprotein A1) and their clinical values.

HbA1C’s high model importance (0.138), despite an insignificant *p*-value (*p* = 0.852), suggests a non-linear relationship with adenoid hyperplasia. Higher HbA1C values in the adenoid hyperplasia group (mean = 7.88 vs. 6.18) indicate possible glycemic dysregulation, which agrees with reports linking metabolic stress and chronic inflammation. The SHAP summary plot revealed that elevated HbA1C levels increased the probability of adenoid hyperplasia, a pattern missed by linear models such as logistic regression (HDL *p* = 0.915, sex *p* = 0.890). This discrepancy highlights XGBoost’s ability to capture complex interactions. However, the high variability (SD = 9.82) and limited sample size (n = 83) for HbA1C necessitate validation in larger cohorts to confirm its clinical relevance.

ALT’s near-significant trend (*p* = 0.082, mean = 26.35 vs. 20.88) and moderate model contribution (0.078) suggest potential liver involvement in adenoid hyperplasia, possibly due to systemic inflammation or subclinical hepatic stress. The swarm plot collage visually confirmed higher ALT values in the adenoid hyperplasia group, supporting its relevance despite lower model importance compared to HDL and HbA1C. The aforesaid finding aligns with studies reporting elevated liver enzyme levels in chronic inflammatory conditions, although adenoid-specific data are sparse [13]. A further exploration of liver function markers (e.g., AST and gamma-glutamyl transferase) can clarify ALT’s role.

The moderate model performance (ROC-AUC = 0.626, recall = 0.556) reflects challenges inherent to the dataset. The small sample size (~20 test samples) and missing data (e.g., HbA1C n = 83, HDL n = 92) possibly reduced the model’s discriminative power, as evidenced by the high cross-validation variability (SD = 0.104). Median imputation mitigated missingness but may have introduced bias, particularly for HbA1C, where high variability obscured statistical significance. Additionally, the slight class imbalance (52 control, 46 adenoid hyperplasia) was addressed with a scale_pos_weight of 1.13. However, low recall suggests missed adenoid hyperplasia cases, which is a concern for clinical practice. The above-mentioned limitations indicate that the model is exploratory rather than definitive, requiring larger datasets for robust generalization.

The negligible contribution of sex (importance = 0.000, logistic regression *p* = 0.890) was expected, given similar distributions across the groups in the count plot. This finding aligns with a relatively balanced epidemiology of adenoid hyperplasia. The other features, such as total cholesterol (*p* = 0.128, importance = 0.060) and LDL (*p* = 0.858, importance = 0.097), had limited predictive values, possibly due to high variability (e.g., total cholesterol SD = 56.23) or overlap between the groups.

SHAP analysis enhanced interpretability, confirming HDL, HbA1C, and fasting blood glucose as key drivers while revealing directional effects (e.g., lower HDL values increasing the probability of adenoid hyperplasia). This transparency is crucial for clinical adoption since it bridges machine learning predictions with biological plausibility. The swarm plot collage provided a visual complement, highlighting the distinct distributions of HDL and ALT and the variability of HbA1C. This reinforced the need for targeted biomarker studies.

The use of 100 trees in the XGBoost model (n_estimators = 100, max_depth = 3, learning_rate = 0.1) may exacerbate overfitting risks given the limited training data, as evidenced by the high cross-validation variability. Generating learning curves, reducing model complexity (e.g., ≤30 trees with stronger regularization such as increased min_child_weight or gamma), and comparing performance with simpler models like logistic regression or LASSO would provide valuable insights into model stability and overfitting. However, these analyses were not feasible within the current study’s timeline and resource constraints. Future work will prioritize these steps, including generating learning curves to assess training and validation performance, optimizing XGBoost with fewer trees and stronger regularization, and benchmarking against logistic regression and LASSO to identify the most robust model. Expanding the dataset (>200 patients) will also enhance training stability and reduce variability, supporting more reliable biomarker identification.

Clinically, the results obtained suggest that routine lipid and glycemic panels could aid adenoid hyperplasia risk stratification, with HDL as a primary candidate for further validation. The unexpected prominence of HbA1C warrants investigation into metabolic–inflammation linkages, potentially guiding screening protocols. Elevated ALT levels highlight the importance of monitoring liver function, particularly in chronic cases. However, the model’s moderate performance precludes immediate diagnostic use, emphasizing the need for larger, multi-center studies to improve predictive accuracy.

Future research should prioritize increasing the sample size (>200 patients) to reduce variability and enhance model stability. Collecting complete biochemical profiles, particularly for HbA1C, would minimize imputation bias. Exploring feature interactions (e.g., HDL*HbA1C) and additional markers (e.g., inflammatory cytokines) could uncover synergistic effects. Finally, comparing XGBoost to simpler models (e.g., logistic regression with HDL and ALT) or ensemble approaches could optimize clinical utility.

## 5. Conclusions

This study provides novel insights into the lipid and metabolic profiles associated with nasopharyngeal adenoid hyperplasia in adults, identifying HDL, HbA1C, and ALT as potential biomarkers. The significant reduction in HDL levels in the adenoid hyperplasia group (mean = 48.68 vs. 51.31, *p* = 0.029, Cohen’s d = −0.156) and its prominence as the top predictor in the XGBoost model (importance = 0.178) suggest that lipid dysregulation, particularly lower HDL, may be linked to chronic inflammation or upper airway obstruction in adenoid hyperplasia. This finding aligns with prior evidence in pediatric adenoid hypertrophy and underscores HDL’s potential as a clinical biomarker for risk stratification. HbA1C, despite its non-significant *p*-value (*p* = 0.852), emerged as a key predictor (importance = 0.138), with higher values in the adenoid hyperplasia group (mean = 7.88 vs. 6.18), indicating possible glycemic dysregulation that warrants further investigation, especially given its high variability (SD = 9.82). The near-significant trend for ALT (mean = 26.35 vs. 20.88, *p* = 0.082, importance = 0.078) suggests subclinical hepatic stress, potentially related to systemic inflammation, highlighting the need for liver function monitoring in these patients. The XGBoost classifier achieved moderate performance (ROC-AUC = 0.626, accuracy = 0.650), with cross-validation revealing variability (mean ROC-AUC = 0.669, SD = 0.104), likely due to the small sample size (n = 98) and missing data (e.g., HbA1C n = 83). These limitations underscore the exploratory nature of the model, which requires validation in larger, multi-center cohorts. Clinically, our findings suggest that routine lipid and glycemic panels, particularly HDL and HbA1C, could aid in identifying at-risk patients, while elevated ALT levels may prompt liver function assessments. Future research should focus on expanding sample sizes (>200 patients), collecting complete biochemical profiles, and exploring additional inflammatory markers (e.g., cytokines) to refine predictive models and enhance the management of adenoid hyperplasia in adults. By leveraging machine learning and SHAP analysis, this study lays a foundation for interdisciplinary approaches bridging otolaryngology and endocrinology, offering a pathway toward improved diagnostic and therapeutic strategies.

## Figures and Tables

**Table 1:** Comparison of biochemical parameters between the adenoid hypertrophy and control groups. Note: A Bonferroni correction was applied (α’ = 0.00167) to control the false-discovery rate for 30 tests (10 biomarkers × 3 tests); no *p*-values met this threshold. Units: HDL cholesterol, LDL cholesterol, total cholesterol, triglycerides, fasting blood glucose (mg/dL); HbA1C (%); CRP (mg/L); ALT, AST (U/L).

Parameter	ANOVA *p*-Value	Kruskal–Wallis *p*-Value	Permutation *p*-Value	Adenoid HyperplasiaMean	Control Mean	Adenoid Hyperplasia	Control n
HDL Cholesterol	0.461	0.029	0.475	48.68	51.31	40	52
ALT	0.084	0.186	0.082	26.35	20.88	46	52
Total Cholesterol	0.128	0.195	0.131	166.67	182.35	42	52
CRP	0.271	0.445	0.306	7.6	14.09	42	46
AST	0.736	0.718	0.752	22.42	23.1	45	51
Fasting Glucose	0.333	0.82	0.332	123.3	113.79	46	52
HbA1C	0.233	0.852	0.195	7.88	6.18	34	49
LDL Cholesterol	0.858	0.853	0.856	107.65	106.33	40	52
Triglyceride	0.651	0.834	0.65	141.6	149.52	42	52

**Table 2:** Descriptive statistics and group comparisons.

Parameter	Adenoid Hyperplasia (Mean ± SD, n)	Control (Mean ± SD, n)	Kruskal–Wallis *p*-Value	Cohen’s d (95% CI)
HDL	48.68 ± 21.87, 40	51.31 ± 11.80, 52	0.029	−0.156 [−0.570, 0.258]
ALT	26.35 ± 16.93, 46	20.88 ± 11.42, 52	0.186 *	0.374 [−0.026, 0.774]
Total Cholesterol	166.67 ± 56.23, 42	182.35 ± 42.85, 52	0.195	−0.316 [−0.727, 0.095]
HbA1C	7.88 ± 9.82, 34	6.18 ± 1.18, 49	0.852	0.256 [−0.180, 0.692]
Fasting Blood Glucose	123.30 ± 77.49, 46	113.79 ± 44.76, 52	0.82	0.147 [−0.248, 0.542]
LDL	107.65 ± 40.37, 40	106.33 ± 34.22, 52	0.853	0.036 [−0.378, 0.450]
Triglycerides	141.60 ± 92.37, 42	149.52 ± 83.47, 52	0.834	−0.090 [−0.500, 0.320]
CRP	7.60 ± 10.23, 42	14.09 ± 18.47, 46	0.445	−0.438 [−0.851, −0.025]
AST	22.42 ± 10.75, 45	23.10 ± 10.51, 51	0.718	−0.064 [−0.463, 0.335]

* Permutation test *p* = 0.082 for ALT.

## Data Availability

The original contributions presented in this study are included in the article. Further inquiries can be directed to the corresponding author.

## Abbreviations

LDL	Low-density lipoprotein
HDL	High-density lipoprotein
HbA1C	Glycated hemoglobin
CRP	C-reactive protein
ALT	Alanine aminotransferase
AST	Aspartate aminotransferase
